# The Effect of Fibrin Sealants on Tubal Reanastomosis: A Comprehensive Review of the Literature

**DOI:** 10.3390/jpm16010012

**Published:** 2025-12-31

**Authors:** Dimitrios Papageorgiou, Vasilios Pergialiotis, Ioakeim Sapantzoglou, Eleni Sivylla Bikouvaraki, Nikolaos Salakos, Stylianos Kykalos, Konstantinos Kontzoglou

**Affiliations:** 1 Department of Gynecology, Athens Naval and Veterans Hospital, 11521 Athens, Greece; 2 Laboratory of Experimental Surgery and Surgical Research “N.S. Christeas”, School of Medicine, National and Kapodistrian University of Athens, 11527 Athens, Greece; 3 Unit of Gynecologic Oncology, First Department of Obstetrics and Gynecology, “Alexandra” Hospital, School of Medicine, National and Kapodistrian University of Athens, 11527 Athens, Greece; 4 School of Medicine, National and Kapodistrian University of Athens, 11527 Athens, Greece; 5 Second Department of Propaedeutic Surgery, “Laikon” General Hospital, School of Medicine, National and Kapodistrian University of Athens, 11527 Athens, Greece

**Keywords:** tubal reanastomosis, fibrin sealant, biological adhesive, fertility outcomes, peritoneal adhesions

## Abstract

**Background/Objectives**: Female tubal factor infertility is a major clinical challenge. While surgical repair of the fallopian tubes remains the traditional standard, biological fibrin sealants have been proposed to reduce tissue trauma and improve reproductive outcomes. **Methods**: We conducted database searches of PubMed/MEDLINE, EMBASE and Google Scholar until 31 August 2025, using the keywords “tubal anastomosis”, “tubal reanastomosis,” “tubal reanastomosis”, “uterine horn anastomosis”, “fibrin glue”, “fibrin sealant”, “biological sealant”, “tissue adhesive”, “rabbit”, “rat” and “sterilization reversal.” Reference lists of retrieved articles have been examined to find studies which tested end-to-end tubal (or small-animal uterine horn) anastomosis through biological adhesives with or without additional components to evaluate patency success, fertility results and adhesion formation. **Results**: Thirteen studies met the inclusion criteria (eleven animal; two human). Rat and rabbit models demonstrated that fibrin sealants with intraluminal splints and one-to-two anchoring sutures produced results comparable to microsutures for patency (tubal patency rates of 75–100%) and pregnancy success (pregnancy rates of 60–83%) while reducing surgical time and decreasing peritubal adhesions. The success rates of the procedures depended on the anastomosis locations. Isthmic–isthmic anastomosis produced better results than ampullary repairs which tended to fail or develop stenosis. Fibrin sealant-only repairs without splinting were associated with lower patency (almost 60%) despite acceptable histologic healing. Human data showed similar pregnancy rates (intrauterine pregnancy in about 40–50% of women) and tubal patency but no consistent decrease in adhesions. Ectopic pregnancy rates ranged from 9 to 11%. **Conclusions**: Fibrin sealants are useful adjuncts to microsurgical tubal repair, but they should not replace the basic repair procedures. The effectiveness of this procedure is dependent on three critical factors: precise segment alignment, proper use of splints and stents, and selection of segments with comparable caliber. In a personalized-medicine framework, fibrin-assisted reanastomosis may offer a tailored option for selected women who desire natural pregnancy. Modern standardized research is required to define indications and analyze how the adaptation of fibrin sealants in minimally invasive procedures affect reproductive outcomes, ectopic pregnancy rates, and adhesion development.

## 1. Introduction

Female tubal factor infertility is a significant clinical issue. Surgical repair of the fallopian tubes via segmental excision and reanastomosis of previously ligated or affected segments remains a viable option for women seeking to reverse sterilization or address localized tubal obstruction. Research conducted demonstrates that tubal reanastomosis results in significant rates of successful spontaneous pregnancies to patients who receive this treatment. The main goal of tubal reanastomosis is to restore tubal continuity that maintains natural mucociliary function while minimizing the formation of peritubal adhesions and intraluminal stenosis. The advancement of Assisted Reproductive Technologies (ART) has introduced new treatment methods, yet microsurgical tubal reanastomosis remains an appropriate selection for selected patients who want to achieve live-birth rates comparable to other fertility treatments. The success of the procedure depends on these factors because they determine both tube’s patency and patients’ chances of preserving fertility and their risk of developing an ectopic pregnancy [1,2,3]. From a personalized medical perspective, the choice between tubal reversal and IVF depends on individual patient characteristics which include their age-related fertility decline, ovarian reserve and semen quality, residual tubal length, previous sterilization method and patient preferences regarding treatment approach and desired pregnancy timeline

The conventional technique for executing tubal reanastomosis employs microsurgical suturing as its primary mechanism. This classic approach requires the use of thin monofilament sutures (8-0 or 10-0), atraumatic tissue handling and stenting or splinting of the lumen to align the cut ends. The objectives are to achieve watertight mucosa-to-mucosa coaptation, preserve tubal width, and minimize serosal trauma to reduce peritubal adhesion development. However, microsuturing is technically demanding, time consuming and it can trigger foreign body reactions which lead to granuloma development, anastomotic narrowing and adhesion formation that impair fertility and increase the risk of ectopic pregnancy [2,3,4].

Biological adhesive-sealant compounds, namely fibrin-based sealants referred to as “fibrin glue,” have been proposed as alternatives to sutures in microsurgical applications. Fibrin sealants are composed of concentrated fibrinogen and thrombin that polymerize to establish a fibrin clot at the site of repair, facilitating mechanical coaptation, hemostasis and providing a temporary framework for healing [5,6]. They are used in many surgical specialties to assist tissue approximation and enhance hemostasis, since their safety and efficacy as topical sealants has been already proved [7,8].

Numerous experimental studies, primarily involving rats, have evaluated fibrin sealants in tubal anastomosis models. These studies investigated both mechanical success and post-surgical adhesion development and evaluated pregnancy rates and outcomes and tissue regeneration of tubal and uterine horn layers. However, the majority of studies about fibrin sealants were performed during a time when surgical platforms and robotic systems were not as advanced as they are today. At the same time, tubal reversal is now offered selectively, in women who strongly desire natural conception or who seek alternative solutions to IVF treatment, due to limited access. The use of any adjunct that shortens surgical duration, minimizes tissue damage and enhances healing requires thorough re-evaluation.

The aim of our review is to summarize the existing experimental and early clinical data on the use of biological adhesive-sealant agents, with an emphasis on fibrin-based sealants, in tubal anastomosis. We assess whether these sealants can accomplish effective tubal restoration, characterized by patency, fertility, and tolerable complication rates, equivalent to traditional microsurgical suturing. We also recognize technological limits and anatomical constraints that must be resolved prior to the confident integration of fibrin sealants into standard fertility-preserving tubal surgery.

## 2. Materials and Methods

**Figure 1:**
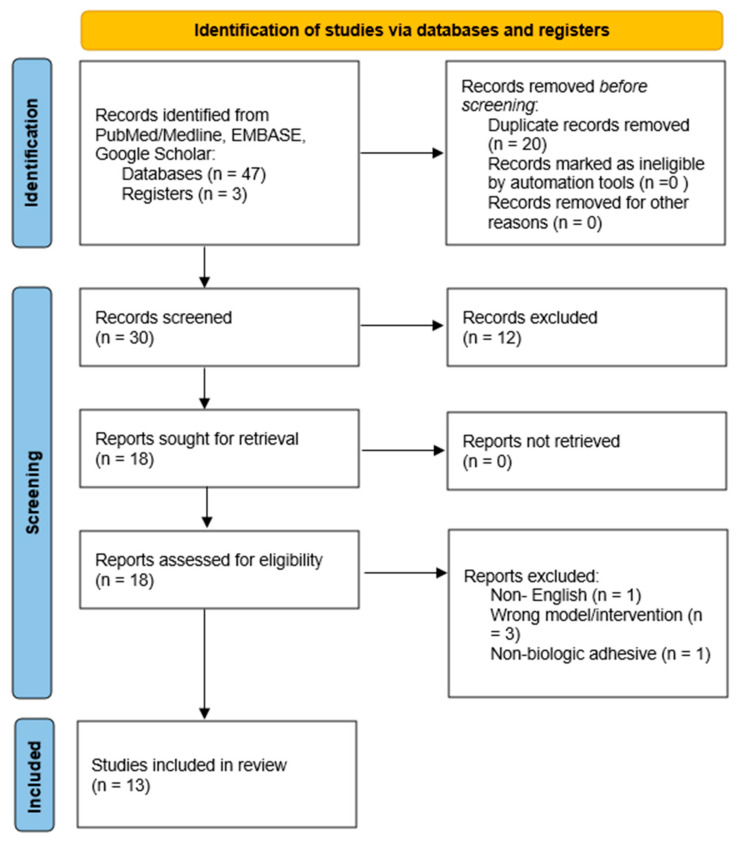
Search strategy flowchart.

We conducted a primary search using the databases EMBASE (1980–2025), Pubmed/Medline (1966–2025) and Google Scholar (2004–2025) in addition to the reference lists of full-text publications that were electronically retrieved. The date of our most recent search was established as 31 August 2025. In PubMed/MEDLINE, our search approach was: (“tubal anastomosis” OR “tubal reanastomosis” OR “uterine horn anastomosis”) AND (“fibrin glue” OR “fibrin sealant” OR “biological sealant” OR “tissue adhesive”) AND (“rabbit” OR “rat” OR “sterilization reversal”). Analogous combinations of these terms were used in EMBASE and Google Scholar. Figure 1 summarizes our search strategy.

Every study that assessed end-to-end anastomosis or reanastomosis of the fallopian tube or its accepted small-animal analogue (rat uterine horn/oviductal segment) and explicitly used a biological adhesive-sealant (e.g., fibrin glue/sealant) either alone or in conjunction with sutures, or compared such sealants with conventional microsurgical suturing and reported at least one relevant outcome such as tubal patency, pregnancy or implantation rates, adhesion formation and histologic healing was deemed qualified for inclusion. The selected articles were required to be original works written in English. Our study included original experimental research. Every other article type has been excluded. We also excluded studies that evaluated fibrin sealants only as hemostatic agents without any description of tubal approximation and research without patency or healing outcomes. The studies were independently evaluated by the authors D.P. and I.S. The reference lists of these papers were examined for any potentially neglected studies.

The search revealed 50 potentially relevant studies, of which 20 were removed due to the presence of irrelevant information. Three authors assessed the abstracts and title pages of all electronic papers to ascertain their eligibility following deduplication. After collecting and reviewing the full copies of articles that were regarded potentially relevant, the decision to include research in the current review was reached. After excluding all other investigations, the current comprehensive review comprised 13 original publications (Figure 1). Table 1 shows the methodological characteristics and the key findings of the included research.

The limited number and high heterogeneity of included studies required us to conduct a qualitative assessment instead of performing a quantitative meta-analysis. The assessment of animal experimental studies included four essential elements (randomization, blinding of outcome assessment, control group size and follow-up completeness). The assessment of human studies included two main factors (clarity of inclusion and exclusion criteria, study design/type and loss of follow up) (Table 2).

## 3. Results

### 3.1. Fertility Preservation and Tubal Patency

#### 3.1.1. Animal Models

Adamyan et al. conducted two essential rat uterine horn studies to evaluate traditional microsurgical techniques with 8-0 Prolene sutures against fibrin glue used as a standalone material or with minimal suturing [19,20]. The study which used 32 rats showed that fibrin glue alone achieved the same success rates as fully sutured repairs for both pregnancy outcomes and lumen patency [19].

A larger study of 63 rats demonstrated that fibrin-glue uterine horn reanastomosis produced 83.3% pregnancy success rates which matched the 80% success rate of microsuture reconstructions without any evidence that fibrin glue harmed fertility [20]. As in the earlier study, pregnancy was confirmed by the presence of implantation sites and embryos in the reconstructed horns at necropsy and patency was evaluated by both macroscopic inspection and dye testing. Both studies demonstrate that fibrin sealant enables successful rat uterine horn reanastomosis which support embryo development and implantation when the horns maintain proper alignment [19,20]. A study which used 20 rabbits demonstrated that fibrin glue (with splinting) for fallopian tube reanastomosis resulted in 100% patency without peritubal adhesions and produced similar fertility outcomes with sutured control groups. The repair site maintained its typical tubal fold architecture and cilia movement according to electron microscopy results [13]. Additionally, Scheidel et al. demonstrated that fibrin-glue reanastomosis in rabbits’ experimental model resulted in patent tubes with subsequent pregnancies, proving feasibility in a real oviduct model [9]. More detailed, the researchers found a tube patency success rate of 75% (30/40 tubes) and 60% pregnancy success (12/20 animals) after the procedure. Dargenio et al. used serial scanning electron microscopy to observe and compare tubes of 8 rabbits following fibrin-glue anastomosis on one side and microsuture anastomosis on the contralateral tube and proved that tubes regained their transport ability through epithelial cell growth and ciliated mucosa regeneration [10]. After mating with proven fertile males six weeks after surgery and the researchers euthanized the animals two weeks later to count corpora lutea and implantation sites for each side. They found no significant differences between sutured and fibrin-glued tubes regarding implantation and pregnancy success and patency rates. Spernol’s et al. experimental research on rabbits demonstrated that fibrin usage leads to complete but ampullary tissue is more sensitive to damage [11].

Overall, sealant-based repair procedures showed variable success across studies. Kamaci et al. conducted a 20-rat study in which the right uterine horn was transected and immediately reanastomosed with either fibrin sealant alone (10 rats) or standard microsurgical suturing (10 rats), with the contralateral horn serving as an internal control. Despite equal adhesion scores and histology outcomes, the fibrin-sealant group achieved 60% patency success whereas the microsurgery group obtained 90%. The study found that fibrin alone does not ensure the establishment of a circular coaxial lumen. Minor rotational errors or local tissue collapse will cause the anastomosis to fail, even if microscopic tissue healing appears to be good [21]. In contrast, some other models used an intraluminal splint or stent to keep the two ends perfectly aligned while the glue dried. Especially, Gauwerky and Klose created the “pelviscopic” rat model using endoscopic tubal anastomoses that required the administration of fibrin glue to an inlaying splint and one seromuscular stitch for the procedure. The tube morphology demonstrated excellent patency with no obstructions or stenosis [14]. The study of Gauwerky et al. found that splinting is necessary when using fibrin glue for treatment, although the splint material can induce mucosal tissue injury [13].

These findings illustrate that biologic fibrin sealants create a sperm-permeable channel that promotes embryo development following tubal disruption. The important distinction is that they also provide short-/mid-term reproductive readouts in controlled animal systems that show that sealant-based approximation can work, making it promising for use in the human reproductive system with equal results. However, it is of utmost importance to mention that biological sealants are bonding chemicals that need stenting or, at the very least, a positioning stitch to attain geometric precision required for long-term patency.

#### 3.1.2. Human Studies

The human evidence regarding fibrin sealant-assisted tubal reanastomosis remains limited because most studies were conducted before the adoption of modern laparoscopic and robotic surgical techniques. The study by Rücker et al. involved 28 women who underwent tubal sterilization reversal using Swolin’s one-stitch technique with fibrin–thrombin adhesive. The researchers achieved 13 intrauterine pregnancies among the 28 women who received at least one year of follow-up. Three ectopic pregnancies and one miscarriage were also documented. The researchers reported the best results in cases of isthmic–isthmic anastomoses because four out of six women became pregnant with intrauterine fetuses [17].

Tulandi performed a retrospective study to evaluate the results of tubal reanastomosis between patients who received only sutures and those who received fibrin sealant at the anastomotic site. The research established that pregnancy success rates and adhesion formation between the two groups showed no significant difference [18]. Those results demonstrated that fibrin sealant functions as an additional tool for microsurgical tubal reanastomosis, yet the findings remain uncertain because of insufficient participant numbers.

The analysis of historical data needs modern microsurgical and laparoscopic tubal reversal success rates to function as current reference points. The present laparoscopic tubal reanastomosis techniques allow 55–75% of patients to get pregnant but only 40–55% of women under 40 who meet specific tubal length criteria and indication standards will have live births. The study by Karayalcin et al. showed that 55.5% of 32-year-old women achieved pregnancy through laparoscopic reanastomosis but 3.7% developed an ectopic pregnancy [22]. The research by Godin et al. showed that pure laparoscopic tubal sterilization reversal resulted in 75.3% pregnancy success and 52.7% delivery success rates [23]. Recent research on microsurgical and laparoscopic reversal procedures documented that these methods produce 70% cumulative pregnancy success for women who meet specific selection criteria which include being under 38 years old and having tubal lengths of 4 cm or more [3,24,25].

In summary, the available human studies about fibrin sealant use in tubal reversal show no adverse effects on patency or pregnancy success but the studies remain underpowered to prove sealant superiority over standard microsurgical techniques. The best available fertility data come from modern suture-based laparoscopic and microsurgical surgical series [1,3,24,25].

### 3.2. Tissue Damage and Peritubal Adhesions

The potential benefit of fibrin sealants in tubal surgery emerges from their ability to minimize tissue damage and foreign body reactions which occur when using multiple stitches for suturing.

#### 3.2.1. Animal Models

In a 1992 rat study, fibrin glue was associated with much fewer adhesions surrounding the anastomotic site than the standard multi-suture microsurgical repair, as well as shorter operation time [19]. The quantity of adhesions influences the outcome since peritubal adhesions can cause the repaired segment to become narrow or develop kinks, resulting in decreased ovum collection and possible partial occlusion, which can lead to fertility issues or ectopic pregnancy. Similar results in rabbit research suggest that fibrin sealants generate local barriers that prevent adhesions from developing [13,26]. Spernol et al. examined rabbit oviducts anastomosed with fibrin adhesive using light and scanning electron microscopy. The researchers observed minimal local inflammation after the procedure while the adhesive material disappeared completely and the anastomotic site showed only minor changes to the epithelial cells [11].

These experimental research findings are consistent with the general surgical literature on fibrin sealants which shows that local fibrin application reduces tissue damage but has not consistently demonstrated a strong independent effect on adhesion prevention [27]. The main factors that determine adhesion formation in gynecologic surgery include the extent of peritoneal injury, ischemia and desiccation and the presence of blood or foreign materials. The selection of closure techniques serves as one part of a complete adhesion prevention strategy [28,29].

#### 3.2.2. Human Studies

On the other hand, early human data produce more reliable results and do not show an increase in peritubal adhesions compared with suturing. Tulandi’s clinical study on women undergoing tubal reanastomosis failed to show a significant decrease in postoperative adhesions when fibrin sealant was used instead of standard suturing [18]. Adhesion biology in the human pelvis is more complex and multifactorial, including bleeding, tissue manipulation, thermal damage and peritoneal desiccation [30,31]. Thus, swapping sutures for glue may not decrease the formation of adhesions postoperatively. The anti-adhesion advantages seen in rat research are less substantial when applied to human patients. That has to do with the fact that in experimental studies rats with fresh healthy tissues undergo surgeries in very controlled and sterile conditions, but humans, on the other hand, frequently present with pelvis pathology such as endometriosis, a history of abdomen or pelvic operations and inflammatory diseases.

Overall, available evidence suggests that fibrin sealants do not cause tissue damage or peritubal adhesion formation and may be neutral or slightly favorable when used within a microsurgical and minimally traumatic technique. The adhesion benefit has potential, but it has not progressed to the point where it can be used as a decisive factor in clinical practice. Robust adhesion data from contemporary human tubal-reversal cohorts are lacking.

### 3.3. Impact of Fibrin Sealants’ Application on Surgical Time and Procedure Complexity

A fundamental subject throughout surgical research is technical simplicity. Adamyan’s et al. rat data report that surgeons obtained effective anastomoses by utilizing fibrin glue, which required less tissue manipulation and a shorter operating time than typical microsurgical approaches [9]. In rabbit experimental models, Scheidel et al. and Dargenio et al. found that surgical oviduct approximation took less time when fibrin glue was used for anastomosis compared with multilayer microsuture techniques [9,10]. Tulandi demonstrated that fibrin sealant application during tubal reanastomosis facilitates a less suture-intensive technique, maintaining anastomotic security, but did not report detailed time metrics [18].

Early human data supports this. Especially, Rücker et al. developed a simplified approach for fallopian tube reversal that involved employing a single stitch and fibrin-thrombin adhesive to seal the anastomosis [17]. The authors utilized one or two seromuscular sutures to connect the tubes before sealing the anastomosis with fibrin glue. In 33 women treated this way (28 with ≥1 year of follow-up), 13 intrauterine pregnancies, three ectopic pregnancies, and one miscarriage were reported. Interestingly, four intrauterine pregnancies occurred in six women. The authors mentioned that this approach saves surgical time without compromising treatment outcomes [17].

Modern human data from laparoscopic and robotic tubal reversal series using microsuturing techniques, mention operative durations that are acceptable in routine practice [1,3,22,23,24,25]. Godin et al. reported mean operative times around one hour for laparoscopic reversal using a four-stitch technique, with good safety and fertility outcomes [23]. These data show that, in experienced hands, sutured laparoscopic anastomosis is technically feasible and does not necessarily require further time-saving measures.

Classic tubal reanastomosis requires very sophisticated microsurgical methods to be performed. The use of fibrin sealants allows surgeons to make stable tissue contact with minimum anchoring stitches, allowing them to perform tubal reversal surgeries even if they lack microsurgical abilities. The use of fibrin sealants may shorten the anastomosis step and reduce technical complexity, particularly for surgeons early in their minimally invasive learning curve. The key difficulty, though, is to reduce time while maintaining success rates of the method, since the healing process of a badly aligned anastomosis, which results in narrower or angulated structures, is less effective than a microsuture repair, which may take longer to complete perfectly.

### 3.4. The Effect of Fibrin Sealants on Different Application Sites

The research of Gauwerky et al. on rabbits showed that surgical results depend on the physical structure of tissues. The diameters of isthmic–isthmic anastomoses stayed unchanged while their folds kept their exact alignment. On the other hand, the wide segments of ampullary–ampullary anastomoses developed virous complications such as fistulas and hydrosalpinx. Intratubal adhesions and stenosis and intraluminal fibrin deposits were, also, reported [12,14]. Although Spernol et al. similarly reported morphological recovery following fibrin reanastomosis, they emphasized that results in the rabbit ampulla are influenced by tissue delicacy and local geometry [11].

Importantly, human tubal reversals are technically easier and more successful when reanastomosis occur on equal diameter segments of the isthmic region, whereas ampullary anastomosis are technically harder and more fragile, due to thinner walls and diameter mismatches [2,32]. The published literature suggests that biologic sealants are efficient for basic isthmic repair procedures, yet surgeons need to use them with reserve when performing complex ampullary reconstruction because they might require additional multiple sutures or stenting [13,14,32].

### 3.5. Impact of Fibrin Sealant Tubal Reanastomosis on Ectopic Pregnancy Rates

The published clinical studies on humans indicate that fibrin sealant could benefit tubal reversal surgery by enhancing fertility results and reducing the chance of ectopic pregnancy.

Rücker et al. monitored 33 women (28 out of 33 for at least 12 months) to document 13 intrauterine pregnancies, three ectopic pregnancies and one miscarriage [17]. The study by Tulandi et al. showed that both methods, sutured tubal anastomosis and fibrin sealant-assisted anastomosis, produced identical results for adhesion development and successful pregnancy achievement. The research demonstrated that fibrin sealant application helps reduce the development of ectopic pregnancies, which pose a major threat to patients undergoing tubal reanastomosis procedures when the reattached segments become misaligned or narrow [18].

These results suggest that fibrin sealant application enables surgeons to perform tissue coaptation with reduced force, which better maintains both tissue size and ciliary function compared to traditional multiple needle insertion methods. Nonetheless, the studies’ initial findings are promising but limited due to their small sample sizes, non-randomized designs, and the fact that they were conducted prior to advancements in laparoscopy and robotic surgery.

In contemporary laparoscopic series, rates of ectopic pregnancy after tubal reversal using suture methods vary between 2% and 7% based on patient age, sterilization method and remaining tubal length. The research shows that laparoscopic tubal anastomosis results in low ectopic pregnancy rates when tubal length is at least 4 cm, bilaterally, and when microsurgical techniques are strictly followed [24].

Overall, the available evidence does not demonstrate a clear correlation between the use of fibrin sealants and ectopic pregnancy rates. The available human studies that used fibrin-assisted techniques remain limited and occurred before modern minimally invasive surgical methods became available. Given that ectopic pregnancy risk is influenced by remaining tubal length, initial tubal disease and surgical technique [1,3,22,23,24,25,32], any independent effect of fibrin sealants is likely to be modest and context-dependent. For now, ectopic pregnancy risk should be regarded as similar, regardless of the technique used for the tubal reanastomosis of the fallopian tubes and patients should follow standard counseling and monitoring protocols.

### 3.6. Minimally Invasive Tubal Anastomosis with Application of Fibrin Sealants

The surgical technique of minimally invasive tubal anastomosis with fibrin sealant shows great potential for reproductive medicine because it enables tubal reversal procedures. Gauwerky and Klose established a uterine horn anastomosis model through their endoscopic procedures on rats, using a training device called “Pelvitrainer”. The researchers implemented a new method by connecting horn segments from the transected area to an intraluminal splint before applying fibrin glue. This procedure was further secured using a single 6-0 seromuscular stitch to fasten the anti-mesenteric border. The post-surgical morphology analyses showed that the reconstructed segments maintained their correct patency, but stenosis or occlusion happened infrequently in particular areas with small tapering [16]. The method provides benefits for upcoming robotic and advanced laparoscopic tubal reversal procedures because it needs minimal suturing in restricted anatomical areas and depends on sealant for stability, which enables surgeons to perform operations through smaller incisions. The system provides multiple benefits, which result in decreased tissue harm and shorter recovery periods for patients.

The surgical environment has changed fundamentally since most fibrin sealant tubal studies were published. High-definition laparoscopy, three-dimensional visualization and robotic platforms have greatly improved visualization and instrument dexterity in pelvic surgery. Multiple recent series show that experienced surgeons can produce equal or better pregnancy rates through laparoscopic and robotic tubal reversal compared to open microsurgery techniques [1,3,22,23,24,25,33,34]. Conventional laparoscopy and robotic systems provide surgeons with stable high-definition images, 3D visualization, tremor reduction and wristed instruments, which improve their ability to perform precise suturing; thus, luminal alignment and serosal approximation are often superior to what is achievable with open surgery [1,3,22,34]. Current research indicates that laparoscopic tubal reanastomosis represents the optimal surgical approach for women under 40 years, because it delivers excellent pregnancy results at affordable costs with short recovery times [1,3].

The endoscopic sealant-based tubal reversal method shows promise but requires precise tube placement through splint or stent insertion and selection of patients who have straight isthmic segments and sufficient length. The experimental data from the present study show that minimally invasive surgical approach can be successfully implemented, but experimental results cannot be verified because there is no current clinical research using human participants that evaluates the application of fibrin sealants during laparoscopic or robotic tubal reanastomosis.

### 3.7. The Role of Biologic Environment on Anastomosis Healing

Schlaff et al. established a rat uterine horn wound-healing model to study how different hormonal statuses affect the mechanical properties of anastomosed horns through measurements of burst strength and extensibility. Researchers discovered that estrogen treatment improved both tensile strength and elasticity during the first two weeks after surgery, but these advantages disappeared between 21 and 42 days [35].

The healing process of an anastomosis depends heavily on biological conditions, which include hormonal changes, tissue injury profile and inflammatory reactions. The success of post-surgical fertility preservation depends on the interaction of these factors with fibrin sealants and sutures [30,31], which in combination create a larger ecosystem that determines patency, adhesions, and healing.

Taken together, the research indicates that fibrin sealants would experience rapid degradation because of the fallopian tube environment, which contains high fibrinolytic activity and hormonal fluctuations. This has several implications for personalized surgical planning. Specifically, when planning individualized treatment, it must be taken into account that sealant formulations containing antifibrinolytic agents may be more suitable for tubal applications, as seen in classic experimental protocols [9,10]. In addition to that, conditions associated with altered peritoneal or tubal biology, such as pelvic inflammatory disease, endometriosis, and previous extensive surgery, may increase the risk of impaired healing and should be factored into individualized decisions regarding the potential benefit of tubal reconstruction vs. IVF [3,28,29,32].

This biological perspective shows that fibrin sealants do not work equally for all patients and require individualized treatment approaches. The use of fibrin sealants should be integrated into a broader personalized treatment strategy that considers patient-specific characteristics, their unique tubal biological environment and medical background.

## 4. Future Directions

Recent studies on surgical results identify three essential priorities, which include operative geometry as the most critical factor over adhesive type selection. The procedure needs surgeons to perform exact coaxial alignment and diameter matching and low-energy hemostasis control through standardized step-by-step procedures that could be performed laparoscopically or robotically [2,3,4]. Future research needs to establish a standardized assessment framework which enables researchers to assess hybrid repair techniques that combine seromuscular sutures with fibrin sealant against standard microsurgical methods while tracking surgeon skills and training development [2,3,4,7].

Secondly, future research should be anatomy-aware. The success of tubal anastomosis depends on three essential factors, which include tubal caliber, remaining length and segment pairing. The surgical choice for patients should involve isthmic–isthmic joins because these anastomoses produce the best results. The upcoming clinical studies need participants to undergo different anastomosis procedures while functional imaging technology assesses both luminal caliber and its patency status because concealed stenosis could result in ectopic pregnancies [2,3,32].

In addition, future research studies should reassess endpoints and perioperative biology status. Tubal reanastomosis results in successful pregnancy and live birth delivery, but different evaluation methods exist to measure the success rates of the different methods [2]. Future cohort studies need to track multiple outcome measures, which include pregnancy duration, successful pregnancy rates and ectopic pregnancy occurrence based on anastomosis type and tubal patency verification through imaging [2,3,32]. They also need to assess how patient age and residual tubal ligation affect surgical and fertility outcomes. Trials need to establish methods which reduce tissue adhesion formation while improving tissue healing through different surgical techniques [7,28,36]. Finally, studies should refine fibrin sealant parameters (viscosity, volume, application plane) and document interactions and possible adverse outcomes in gynecologic minimally invasive surgeries, where fibrin sealants are already used for hemostasis [7,37,38,39].

## 5. Conclusions

Research has proved that tubal reanastomosis enables fertility preservation in particular patients who achieve pregnancy and live birth rates similar to other options, such as in vitro fertilization, with acceptable ectopic pregnancy risks when surgeons with experience perform the procedure. In this context, fibrin sealants should be addressed as complementary adjuncts to microsurgical techniques instead of replacing them, because sutures achieve exact tissue alignment with no tension, and fibrin sealants enhance hemostasis and minimize additional tissue trauma.

Studies on hybrid protocols (use of fibrin sealants combined with microsuturing techniques) in minimally invasive cohorts will enable sealant-assisted tubal reanastomosis to advance from its current status as a promising method into an evidence-based treatment for specific patient groups.

## Figures and Tables

**Table 1:** Methodological characteristics and key findings of included studies.

Study (Authors, Year)	Type of Study	Subjects (n) and Species	Type of Studied Specimen	Anastomosis Site	Sealant/Technique Used	Definition of Patency/Outcome Assessment	Follow-Up Duration	Key Findings
Scheidel et al., 1982 [9]	Original/Experimental	20 New Zealand White rabbits	Rabbit oviduct	End-to-end after 1–2 cm isthmic resection	Allogenic fibrin glue (biologic tissue adhesive)	Patency assessed at necropsy by inspection and probing/contrast of the tubal lumen; functional fertility assessed by standardized artificial insemination followed by pregnancy and delivery. Pregnancy diagnosed by laparoscopy 10–12 days after insemination.	Artificial insemination started 4 weeks after anastomosis; if no pregnancy occurred, insemination was repeated up to three more times (total of four attempts). Animals with four ineffective inseminations were sacrificed. Those that conceived were followed through pregnancy and delivery. Fibrin remnants could be detected histologically up to 50 days post-op, but not beyond. Overall functional follow-up therefore extended from about 4 weeks to ≈3–4 months post-anastomosis.	Patency rate 75% (30/40 tubes) and pregnancy rate 60% (12/20 animals). No spontaneous recanalization or pregnancies in controls. Histology showed only mild–moderate tissue reaction, comparable to microsutures.
Dargenio et al., 1986 [10]	Original/Experimental	8 rabbits	Rabbit oviduct	End-to-end (right 10-0 nylon vs. left fibrin)	Fibrin glue Tissucol^®^—like vs. 10-0 nylon sutures	Patency and fertility evaluated by separate counts of corpora lutea and gestational sacs on each side. A side-specific nidation index (implantations/corpora lutea) calculated. Macroscopic adhesions graded at re-laparotomy.	Animals mated 6 weeks post-surgery and euthanized 2 weeks later (≈8 weeks total).	No significant differences between fibrin-glued and sutured sides in pregnancy rate, nidation index, or patency. Adhesion formation not increased with fibrin. Anastomosis time reduced with glue.
Spernol et al., 1984 [11]	Original/Experimental	26 rabbits	Rabbit oviduct	End-to-end	Fibrin tissue adhesive	Main outcome was morphologic healing at the anastomotic site assessed by light microscopy and scanning electron microscopy: epithelial continuity, ciliation, inflammatory reaction.	Single morphological time-point 3 months after surgery.	Minimal local reaction, no fibrin clots, minimal inflammation. Fewer ciliated cells on SEM. Overall changes comparable to microsutures.
Dargenio et al., 1988 [12]	Original/Experimental	18 New Zealand White rabbits	Rabbit oviduct	End-to-end	Fibrin tissue adhesive (Tissucol^®^-like)	Morphologic healing of tubal mucosa and lumen assessed by SEM: de-epithelialization, appearance of cells with microvilli, onset of ciliogenesis, degree of luminal stenosis and restoration of normal ciliated epithelium. No pregnancy/functional outcomes.	Animals were sacrificed at different intervals between 2 and 56 days after anastomosis: at 2–12, 14, 28, 42 and 56 days. Thus, follow-up spans 2–56 days postoperatively, with maximum follow-up at 8 weeks.	Early (first 4 days) mucosal trauma with de-epithelialization. Cells with microvilli visible by day 6. First cilia by day 10; complete ciliogenesis between 2 and 4 weeks. By 56 days the epithelium resembled normal tube. Healing time with fibrin glue appeared similar to that seen with sutures.
Gauwerky et al., 1988 [13]	Original/Experimental	20 New Zealand White rabbits	Rabbit oviduct	Reanastomosis (various: tubocornual, ampullary-ampullary, isthmic-ampullary)	Fibrin glue vs. conventional microsutures. Intraluminal splint in glue groups	Patency and function assessed at second laparotomy: mechanical patency of all oviducts recorded. Fertility evaluated by number of corpora lutea, implantations, and nidation index for each side. Presence of intraperitoneal adhesions scored.	Patency and fertilityassessed at re-exploration 6–12 weeks after anastomosis.	All reanastomosed oviducts were patent. No intraperitoneal adhesions observed. Fertility (nidation index, pregnancy rate) in isthmic anastomoses was comparable between fibrin and sutures. Ampullary–ampullary and diameter-mismatched anastomoses performed worse with fibrin alone, suggesting glue is less suitable where luminal widths differ significantly.
Gauwerky et al., 1990 [14]	Original/Experimental	20 female Wistar rats	Rat uterine horn	Endoscopic (pelviscopic) horn anastomosis; 6-0 seromuscular stitch + splint + glue	Fibrin glue with one 6-0 seromuscular suture	Anatomical patency and healing of the anastomosed horn assessed by light microscopy and SEM: lumen continuity, absence of stenosis/occlusion, smoothness of edges. No fertility or pregnancy testing.	The uterus and adnexa were perfused and removed 4 weeks after surgery for morphological evaluation. Some SEM images illustrate specimens up to 73 days, but the planned core follow-up is 4 weeks post-anastomosis.	All anastomoses showed good patency and tissue continuity at 4 weeks. Only two of 20 specimens showed tapering of the uterine wall adjacent to the anastomosis. Study demonstrates feasibility of an endoscopic glue-assisted anastomosis model.
Gauwerky et al., 1992 [15]	Original/Experimental	41 New Zealand White rabbits (3 groups)	Rabbit oviduct	(i) Isthmic without resection (ii) Ampullary (iii) Isthmic after resection	Fibrin glue vs. conventional microsutures	Fertility assessed by number of ovulations, number of implantations, and nidation index for each side. Patency verified intraoperatively at laparotomy. Adhesions graded macroscopically around the tubes.	Animals were mated with a proven fertile buck 4–12 weeks after surgery (mean 78.2 days). When pregnancy occurred (palpation of abdominal wall), laparotomy was performed to evaluate ovulations, implantations, patency and adhesions. If no pregnancy occurred, mating was repeated up to three times. Follow-up therefore extended from 4 to 12 weeks + up to three mating cycles until laparotomy at pregnancy or study end.	In isthmic anastomoses (with or without resection), fertility and patency were similar for fibrin-glued and sutured sides. Adhesions were absent or minimal. Ampullary anastomoses had a slightly lower nidation index in both techniques, and gluing was associated with occasional occlusion/hydrosalpinx, suggesting limitations of fibrin glue in ampullary repairs.
Gauwerky et al., 1993 [16]	Original/Experimental	41 New Zealand White rabbits	Rabbit oviduct	Reanastomosis (including disparate lumens)	Fibrin glue vs. conventional microsutures	Morphologic outcomes examined by light microscopy and SEM: mucosal fold alignment, continuity of tubal wall, luminal diameter, presence of polypoid/tubular structures, intraluminal adhesions or stenosis at the anastomosis. No fertility outcomes reported.	Perfusion-fixation and removal of tubes occurred on average 92 days after anastomosis (range 30–202 days). Thus, morphologic follow-up spans 1 to ~6.5 months, with a mean of about 3 months	Detailed morphology: normal mucosal fold pattern and ciliation under optimal conditions. Reiterates risk when joining segments of different caliber/thickness (ampullary).
Rücker et al., 1988 [17]	Human Cohort (Technique report + Outcomes)	33 women undergoing reversal of tubal ligation	Human fallopian tube (sterilization reversal)	Tubo-tubal “Swolin one-stitch” with adjunctive glue	Swolin one-stitch microsurgical technique plus fibrin–thrombin adhesive	Technical success evaluated intraoperatively. Functional outcomes defined as intrauterine pregnancy, ectopic pregnancy and miscarriage after reanastomosis. Routine HSG not systematically reported. Patency essentially inferred from subsequent pregnancies	A follow-up period of at least 1 year was available in 28 of 33 patients. Pregnancy outcomes (intrauterine, ectopic, miscarriage) reported over this ≥12-month period. Maximum follow-up exceeds one year but is not precisely stated.	Among 28 women with ≥1-year follow-up, there were 13 intrauterine pregnancies, three ectopic pregnancies and one miscarriage. In the subgroup with isthmic–isthmic anastomosis, 4 intrauterine pregnancies occurred in 6 patients. Authors concluded that one-stitch + fibrin adhesive can shorten operating time without reducing pregnancy rates
Tulandi, 1991 [18]	Retrospective Study	Study 1: 12 women. Study 2: 28 women (microsurgical tubal reversal)	Human fallopian tube	Tubo-tubal	Fibrin sealant (Tisseel^®^) as adjunct vs. sutures only	Study 1: second-look laparoscopy used to assess adhesions (AFS score) and chromopertubation with methylene blue to test tubal patency at both sides. Study 2: clinical pregnancy outcomes (intrauterine, ectopic) compared between fibrin and non-fibrin groups; cumulative conception probability plotted over time.	Study 1: all 12 women had second-look laparoscopy 6–12 weeks after initial operation, at which adhesions and tubal patency were evaluated. Study 2: cumulative pregnancy probabilities reported at 12 and 18 months post-surgery.	In Study 1, there were no significant differences in adhesion formation or patency between fibrin-treated and sutured sides. In Study 2, cumulative pregnancy rates at 12 and 18 months were similar between groups, but ectopic pregnancies occurred only in the suture-only group. Authors suggest fibrin sealant does not worsen adhesions and may reduce ectopic pregnancy risk, though numbers are small.
Adamyan et al., 1992 [19]	Original/Experimental	32 rats (3 groups)	Rat uterine horn	End-to-end horn anastomosis	Fibrin glue FK-1 vs. 8-0 Prolene sutures vs. combined glue + sutures	Patency and fertility assessed by presence and number of embryos in each horn at sacrifice after mating; macroscopic adhesions at the anastomotic site were scored. Operative time compared among groups.	After healing, rats were mated and then sacrificed at the end of gestation. Uterine horns examined for embryos and adhesions. The paper reports follow-up until completion of one pregnancy after horn reconstruction. The exact number of postoperative days to sacrifice is not specified but corresponds to one full rat gestation after the postoperative waiting period.	Pregnancy and patency comparable between glue and sutures. Significantly fewer adhesions and shorter surgical time with fibrin sealent.
Adamyan et al., 1994 [20]	Original/Experimental	63 rats	Rat uterine horn	End-to-end horn anastomosis	Different biological glues (e.g., Beriplast^®^, FK-1) vs. various microsuture materials	Uterine horn patency and adhesion formation assessed at necropsy. Reproductive function evaluated by pregnancy rates and embryonal features after mating. Patency essentially defined by the ability of the reconstructed horn to sustain embryos.	Animals were mated after healing and sacrificed at the end of gestation, at which time reproductive and adhesion outcomes were assessed. Follow-up therefore extends to one completed pregnancy after surgery. The authors do not specify the exact number of days from anastomosis to sacrifice but it corresponds to the postoperative healing period plus one gestation.	No significant differences in pregnancy rates between microsuture and sutureless glue-based anastomoses (around 80–83%). Glue techniques did not increase adhesions and were considered effective alternatives for reconstructive uterine-horn surgery.
Kamaci et al., 1997 [21]	Original/Experimental	20 Sprague–Dawley rats	Rat uterine horn	End-to-end uterine horn anastomosis	Right uterine horn of each rat transected; animals randomly allocated to group A (Tissel^®^fi) or group B (microsurgical sutures) for reconstruction of the right horn; left horn left intact as internal control. Anastomosis constructed either with fibrin glue alone (no sutures) or with interrupted 10-0 nylon microsutures; no intraluminal stents used.	At 30 days post-op, patency of the reconstructed horn assessed with methylene blue injection via the uterine artery; peri-anastomotic adhesions scored using a semiquantitative scale; healing graded histologically and by SEM (mucosal regeneration, muscularis disruption).	Evaluation performed at 30 days after surgery	Authors concluded that fibrin sealant can be an alternative to microsurgery with similar morphologic results, but given slightly lower patency and no clear advantage, they considered it premature to recommend routine human use based solely on this animal model.

Abbreviations: cm: centimeter, SEM: Scanning electron microscopy, HSG: Hysterosalpingography, AFS: American Fertility Society (adhesion scoring system).

**Table 2:** Qualitative assessment of included studies.

Study (Authors, Year)	Randomization	Blinding of Outcome	Control/Comparison Group	Main Outcomes Assessed	Follow-Up Completeness	Overall Risk of Bias *
Scheidel et al., 1982 [9]	NR	NR	Partial	Patency at necropsy. Fertility (pregnancy after artificial insemination). Histology. Fibrin persistence	High	Moderate-high
Dargenio et al., 1986 [10]	NR	NR	Yes	Patency and fertility via side-specific nidation index. Adhesions. Local histology	High	Moderate
Spernol et al., 1984 [11]	NR	NR	No formal suture control within same experiment. Mainly descriptive	Healing, tissue reaction around glue	High	High
Dargenio et al., 1988 [12]	NR	NR	No concurrent suture control in same protocol. Comparison mainly with historical sutured data	Re-epithelialization, histology outcomes	High	Moderate-high
Gauwerky et al., 1988 [13]	NR	NR	Partial	Patency, fertility, basic morphology	High	Moderate-high
Gauwerky et al., 1990 [14]	NR	NR	No true sutured control group in same minimally invasive model (focus on feasibility)	Patency, lumen continuity, morphology, technical feasibility	High	High
Gauwerky et al., 1992 [15]	“Random allocation” by side mentioned, but details limited	NR	Yes	Pregnancy, adhesions, morphology	High	Moderate
Gauwerky et al., 1993 [16]	NR	NR	Comparative morphology of glued vs. sutured anastomoses at different sites, but not designed as independent controlled trial	Morphology of anastomotic site, lumen configuration	High	Moderate-high
Rücker et al., 1988 [17]	NR	NR	No	Intrauterine and ectopic pregnancy, miscarriage, operative time	Moderate-high	High
Tulandi., 1991 [18]	NR	NR	Study 1: within-patient side-by-side comparison (fibrin vs. sutures on contralateral tubes). Study 2: retrospective comparison of patients with vs. without fibrin sealant	Study 1: second-look adhesions (AFS score) and chromopertubation patency at 6–12 weeks. Study 2: cumulative pregnancy and ectopic rates at 12 and 18 months	Moderate	Moderate-high
Adamyan et al., 1992 [19]	NR	NR	Yes	Pregnancy rates, patency, adhesions, operative time	High	Moderate
Adamyan et al., 1994 [20]	NR	NR	Yes	Pregnancy rates, patency	High	Moderate
Kamaci et al., 1997 [21]	Yes	NR	Yes	Patency at 30 days (methylene blue test). Peri-anastomotic adhesion scores. Histologic and SEM healing	High	Moderate

* Across animal experiments, none reported randomization or blinding, so risk of bias was generally moderate to moderate-high. Human data consisted of small, non-randomized series or retrospective cohorts, leading to overall moderate-high to high risk of bias. Abbreviations: NR: not reported, AFS: American Fertility Society (adhesion scoring system).

## Data Availability

No new data were created or analyzed in this study. Data sharing is not applicable to this article.

## Abbreviations

The following abbreviations are used in this manuscript:

cm	Centimeter
IUP	Intrauterine pregnancy
IVF	In vitro fertilization
SEM	Scanning electron microscopy
AFM	American Fertility Society
HSG	Hysterosalpingography
